# Attenuation of hyperglycemia and amadori products by aminoguanidine in alloxan-diabetic rabbits occurs via enhancement in antioxidant defenses and control of stress

**DOI:** 10.1371/journal.pone.0262233

**Published:** 2022-01-05

**Authors:** Binish Arif, Zarina Arif, Jamal Ahmad, Kahkashan Perveen, Najat A. Bukhari, Jalaluddin M. Ashraf, Moinuddin, Khursheed Alam

**Affiliations:** 1 Department of Clinical Biochemistry, Sher-i-Kashmir Institute of Medical Sciences, Soura, Srinagar, J&K, India; 2 Department of Biochemistry, J.N. Medical College, Faculty of Medicine, Aligarh Muslim University, Aligarh, UP, India; 3 Formerly at Rajiv Gandhi Centre for Diabetes and Endocrinology, J.N. Medical College, Faculty of Medicine, Aligarh Muslim University, Aligarh, UP, India; 4 Department of Botany and Microbiology, College of Science, King Saud University, Riyadh, Kingdom of Saudi Arabia; 5 Department of Clinical Biochemistry, Faculty of Applied Medical Sciences, Jazan University, Kingdom of Saudi Arabia; Stellenbosch University, SOUTH AFRICA

## Abstract

The micro- and macro-complications in diabetes mellitus (DM) mainly arise from the damage induced by Amadori and advanced glycation end products, as well as the released free radicals. The primary goal of DM treatment is to reduce the risk of micro- and macro-complications. In this study, we looked at the efficacy of aminoguanidine (AG) to prevent the production of early glycation products in alloxan-diabetic rabbits. Type1 DM was induced in rabbits by a single intravenous injection of alloxan (90 mg/kg body weight). Another group of rabbits was pre-treated with AG (100 mg/kg body weight) prior to alloxan injection; this was followed by weekly treatment with 100 mg/kg of AG for eight weeks. Glucose, insulin, and early glycation products (HbA1_C_ and fructosamine) were measured in control, diabetic and AG treated diabetic rabbits. The effects of hyperglycemia on superoxide dismutase (SOD), catalase (CAT), glutathione peroxidase (Gpx), reduced glutathione (rGSH), nitric oxide, lipid peroxides, and protein carbonyl were investigated. Alloxan-diabetic rabbits had lower levels of SOD, CAT, Gpx, and rGSH than control rabbits. Nitric oxide levels were considerably greater. AG administration restored the activities of SOD, CAT, Gpx enzymes up to 70–80% and ameliorated the nitric oxide production. HbA1c and fructosamine levels were considerably lower in AG-treated diabetic rabbits. The observed control of hyperglycemia and amadori adducts in alloxan-diabetic rabbits by AG may be attributed to decrease of stress and restoration of antioxidant defenses.

## Introduction

Hyperglycemia can accelerate non-enzymatic glycation of moving and tissue-fixed proteins, which can lead to secondary problems in diabetes mellitus (DM) [1, 2]. The incidence of microvascular (nephropathy, retinopathy, neuropathy, and lower extremity amputations) and macrovascular (cardiovascular) problems may be directly influenced by the poor management of DM [3]. The outcome of glycation is chemical modification of proteins’ amino groups by reactive sugars, carbonyls, etc. Early glycation results in Amadori products. Only a small part of the Amadori-products undergo further irreversible chemical reactions leading to the formation of advanced glycation endproducts (AGEs). Excessive formation of early glycation products may adversely affect diabetic complications [4, 5]. The concentration of Amadori-proteins is at least 2%, whereas the concentrations of AGE-equivalents are less than 0.01% of serum proteins [6]. Although Amadori-proteins are the major representative of glycation proteins’ family, most studies conducted so far have focused on the role of AGEs in diabetes-related complications. Data accumulated so far suggest that Amadori-proteins may play a critical role in the pathogenesis of diabetic complications [7].

During hyperglycemia, Amadori-modification is accelerated [8] and reactive oxygen species (ROS) are produced in large quantities [9, 10]. Furthermore, the onset of DM and related complications has been attributed to excessive generation of ROS [11]. Since, hyper-glycation increases oxidative stress, glycation and oxidation appear to be linked inextricably. The defense line of an organism against ROS includes scavenger enzymes such as superoxide dismutase (SOD), catalase (CAT) and glutathione peroxidase (Gpx). It has been shown that SOD is inactivated by glycation [12]. Studies carried out on aminoguanidine (AG) have revealed that it is effective against AGE formation via its antioxidant action [13, 14]. Pharmacological prevention of Amadori formation is an attractive means of preventing diabetic microvascular complications because it bypasses the usually difficult goal of achieving euglycemia. Thus, AG may prove beneficial in controlling hyperglycemia related complications. Aminoguanidine (pimagedine, CH_6_N_4_), a prototype agent, acts by trapping or scavenging reactive carbonyl intermediates generated by the Maillard reaction, mainly hydroxyaldehydes and dicarbonyl compounds, including methylglyoxal [15, 16]. It is a highly reactive nucleophilic agent that reacts with many biological macromolecules (pyridoxal phosphate, pyruvate, glucose, malondialdehyde, and others). AG prevents arterial stiffening and cardiac hypertrophy in streptozotocin-diabetic rats [17]. Aminoguanidine also protects vascular endothelial cells (ECs) from glyoxal-induced cytotoxicity, barrier dysfunction, and angiogenesis inhibition [18]. It also ameliorates the DM associated cardiac fibrosis [19]. Furthermore, anti-AGE drugs may stimulate endogenous systems engaged in AGE detoxification, such as the AGE-receptor family (AGE-R) and the glyoxalase system [20]. The glyoxalase system detoxifies methylglyoxal, which is converted to S-D-lactoyl glutathione by glyoxalase 1 (GLO1) and then to D-lactate by GLO2 [21]. In the present study, we investigated the effect of aminogianidine on some glycoxidative parameters of alloxan-diabetic rabbits. Alloxan, a chemical diabetogen, is reduced in presence of glutathione (via the alloxan radical) into dialuric acid producing ROS that destroys beta-cells in islets of Langerhans [22]. Levels of HbA_1c_ and fructosamine have been estimated as markers of Amadori products.

## Materials and methods

Healthy albino rabbits (average weight 1.2±0.45 kg) were obtained from the central animal house facility, Jawaharlal Nehru Medical College, Aligarh Muslim University, India. Prior to use, the rabbits were acclimatized for a week. The animals were housed in cages with wide square mesh at the bottom to avoid coprophagy and kept in a well ventilated room maintained at 12 h light/ 12 h dark cycle, 50% humidity and 28±2°C temperature. Eighteen rabbits were divided into three groups: (1) C: control, (2) D: diabetic, and (3) D+AG. All animals received a standard diet and water ad libitum. The protocol was duly approved by the Institutional Animal Ethics Committee, Faculty of Medicine, Aligarh Muslim University, India.

### Induction of diabetes mellitus and experimental design

To induce the experimental type1 DM, the rabbits were fasted for 12 h before giving an intravenous injection of alloxan (Sigma-Aldrich, St. Louis, MO, USA) proportionate to their body mass (90 mg/kg) dissolved in 0.9% (w/v) saline [23]. The glycemia was measured for 8 weeks by the glucose oxidase method using a commercial kit (Accu-check G Mannheim, Germany). All other biochemicals were also measured on weekly basis.

Group 1-Control: injected with 0.9% (w/v) saline.

Group 2- Diabetes mellitus- Intravenous injection of alloxan in saline.

Group 3-Pretreatment with one dose of AG in saline (100 mg/kg) on the day of alloxan injection (i.p.) and then eight weekly doses of 100 mg/kg each of AG. The above dose of AG was selected based on its successful use by other researchers [17, 24].

Blood samples (1–3 ml) were collected from the marginal ear vein of the rabbits on weekly basis for eight weeks and transferred into glass vials devoid of anticoagulant. Sera were separated and stored at -20°C.

At the end of treatments, rabbits were anesthetized with ether/chloroform. The pancreas was excised, snap frozen, and stored at -80°C. Paraffin blocks of the pancreatic sections were prepared, sectioned and mounted on glass slides.

### Measurement of insulin

Insulin was measured in rabbits’ sera by radioimmunoassay using commercial kit (Insulin IRMA Kit, Inc., Immunotech).

### Measurement of HbA_1_c

HbA_1_c concentration in blood samples was determined by HPLC (Bio-Rad D-10^TM^ USA).

### Fructosamine

The serum fructosamine was determined by NBT assay [25]. Briefly, serum samples (200μl) were added to 96-well microtiter plates in duplicate. 100μl NBT reagent (250 μmol/l in 0.1mol/l carbonate-bicarbonate buffer, pH, 10.35; SRL chemicals, India) was added to each well and incubated for 2 h at 37°C. The color was read at 525 nm. The purple color monoformazan was quantitated using an extinction coefficient of 12640 cm^-1^mol^-1^ [26].

### Superoxide dismutase

SOD activity was estimated by the method of Marklund and Marklund [27]. Briefly, serum samples (50 μl) were mixed with 0.1 ml of 8 mM pyrogalllol (SRL chemicals, India) in 50 mM Tris-succinate buffer (pH 8.2). A reference blank was prepared separately by mixing 50 μl of distilled water in the above reaction mixture. The change in absorbance at 420 nm was recorded every 30 sec for 3 min. SOD activity was expressed as units/mg protein.

### Catalase

Catalase was estimated as described earlier [28]. Briefly, the assay mixture consisted of 50 μl of serum samples and 1.0 ml of 30 mM H_2_O_2_ and 1.95 ml of 50 mM phosphate buffer, pH 7.0. The change in absorbance at 240 nm was recorded every 30 sec for 2 min and results were expressed as micromoles of H_2_O_2_ decomposed per minute per mg protein.

### Glutathione peroxidase

Gpx levels in samples were estimated as described earlier [29]. Briefly, in an assay volume of 3 ml, 100 μl of serum was mixed with 1 mM EDTA, 2.4 U/ml glutathione reductase and 10 mM glutathione in 20 mM potassium phosphate buffer pH 7.4. The mixture was incubated for 10 min at 37°C. After incubation, 0.19 mM of NADPH (Sigma Chemical Company, St. Louis, MO, USA) and 12 mM t-butyl hydroperoxide was added to the assay mixture. The decrease in absorbance was measured at 340 nm for 3 min. Gpx activity was expressed as units/mg protein.

### Glutathione

Reduced glutathione (rGSH) was estimated by DTNB method [30]. Briefly, one ml serum sample was precipitated with 1 ml of 4% sulphosalicylic acid (SRL chemicals, India) and cold digested for 1 h at 4°C. The samples were then centrifuged at 1200 g for 15 min at 4°C and supernatant was separated. To 0.4 ml of the supernatant, 2.2 ml of phosphate buffer (0.1 mM, pH 8.1) and 0.4 ml of 3 mM DTNB was added. The yellow color was measured at 412 nm and the amount of reduced glutathione was calculated using the molar extinction co-efficient of 1.36 × 10^4^ cm^-1^mol^-1^.

### Malondialdehyde

Concentration of malondialdehyde in serum samples was estimated as TBARS [31, 32]. Briefly, 10 μl of 1 mM EDTA was added to 500 μl of serum and mixed with 1 ml of 15% (w/v) trichloroacetic acid, followed by 10 min centrifugation at 3000 x g to facilitate protein precipitation. The supernatant was then treated with 200 μl of 1% (w/v) thiobarbituric acid (SRL chemicals, India) and immersed in a boiling water bath for 60 min. After cooling, the resulting chromogen was extracted by adding 600 μl of the sample to 600 μl of n-butanol and centrifuged at 900 x g for 10 min. Finally, 250 μl was removed from the butanolic phase and absorbance was read at 535 nm. The concentration of TBARS was calculated using the molar extinction coefficient of 1.56*×*10^5^ cm^-1^mol^-1^ [33].

### Protein carbonyl

The carbonyl content of serum was determined by the Levine et al. method [34]. Serum samples (100 μl) were precipitated with ice-cold TCA (10%; v/v) and kept at 4°C for 10 min; the samples were centrifuged for 3 min at 11000 x g. The pellets were resuspended in 500 μl of 2 M hydrochloric acid with 10 mM 2,4-dinitrophenylhydrazine (DNPH; Merck, Darmstadt, Germany). The samples were then vortexed for 1 h at room temperature before being precipitated with 0.5 ml of TCA (20%; v/v) and centrifuged for 3 min at 11000 x g. The pellets were rinsed three times with 1.0 ml of ethanol/ethyl acetate (1:1) after centrifugation to eliminate any DNPH. Finally, 1 ml of 6 M guanidine hydrochloride (SRL chemicals, India) diluted in 20 mM potassium phosphate buffer (adjusted to pH 2.3 with trifluoroacetic acid) was used to suspend the protein pellet. The absorbance was measured at 360 nm and the concentration of protein carbonyl was determined using a molar absorption coefficient of 22,000 cm-1mol-1 and represented as nmol/mg protein.

### Nitrite

Serum nitrite concentration was estimated using Griess reagent (1 g/l sulfanilamide, 25 g/l phosphoric acid, and 0.1 g/l *N*-1-naphthylethylenediamine dihydrochloride (Sigma Chemical Company, St. Louis, MO, USA) according to a previously described method [35]. Briefly, serum samples (200 μl) were deproteinized with 40% zinc sulphate solution. The samples were vortexed and incubated for 15 min at room temperature. After incubation, the samples were centrifuged for 5 min at 3000 x g. Hundred μl each of the supernatant and Griess reagent, respectively was added in duplicate to 96-well microtiter plates and incubated for 10 min at 37°C. The absorbance of the purple color was measured at 540 nm. Sodium nitrite was used as standard to determine nitrite content.

### Light microscopy

At the end of study period rabbits were sacrificed by exsanguinations and pancreas was removed carefully. The organ was washed with 0.9% (w/v) saline and fixed in 10% buffered formalin overnight. It was then dehydrated with increasing strength of ethanol, cleared in xylene and wax impregnated before finally casting and blocking. Paraffin blocks of the pancreas were cut into 5 μm thick sections and de-waxed on hot plate and hydrated using descending grades of ethyl alcohol. Haematoxylin (nuclei stain) and eosin (cytoplasmic stain) were used. The sections were again dehydrated with ascending grades of ethyl alcohol before finally mounting on DPX for observation under the microscope.

### Statistical analysis

Kolmogorov-Smirnov test of normality showed that the data were normally distributed. Values are expressed as mean ± SD. Student’s t-test (SPSS) was employed to compare two groups at a time. A p-value < 0.05 was considered statistically significant.

## Results

### Monitoring of glucose and insulin

**Fig 1:**
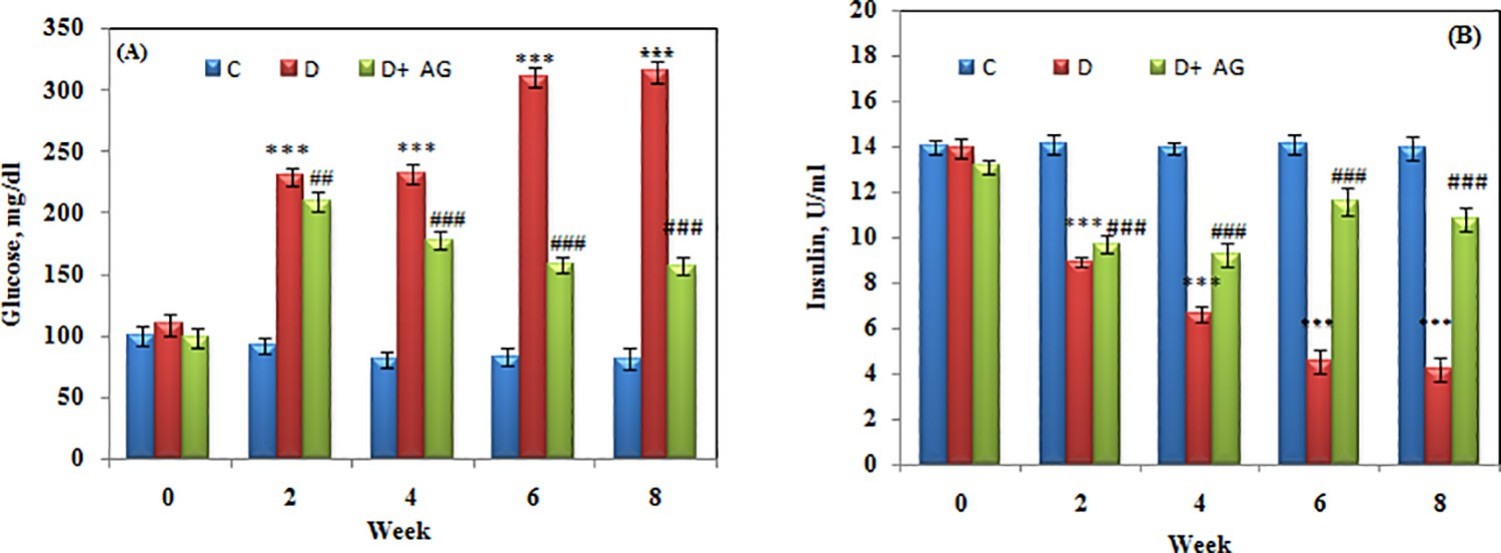
Glucose (**A**) and insulin levels (**B**) of alloxan-induced diabetic rabbits treated for 8 weeks with aminoguanidine. C = Control; D = Diabetic; AG = aminoguanidine. Values are mean ± SD, (n = 6 rabbits/group). *** Significant difference (p<0.0001) as compare to control. ### Significant difference (p<0.0001) as compare to diabetic rats.

Before treatment, there was no discernible change in the basal blood glucose levels of the animals in the three groups. When compared to the control group, the rabbits had a significant (p< 0.05) increase in blood glucose and a drop in insulin levels after a single injection of alloxan (Fig 1A and 1B). Furthermore, alloxan-injected rabbits remained hyperglycemic for the duration of the study. Alloxan-induced hyperglycemia was observed to be considerably lower in AG-treated diabetic rabbits; serum insulin level was found to be significantly restored.

### HbA_1_c and fructosamine

**Fig 2:**
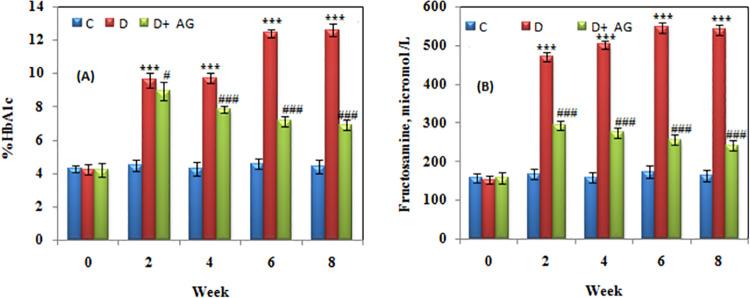
Early glycation markers (Amadori products) of alloxan-induced diabetic rabbits treated for 8 weeks with aminoguanidine. Panel (**A**) is % HbA_1c_ and Panel (**B**) is fructosamine. Values are mean ± SD, (n = 6 rabbits/group). *** Significant difference (p<0.0001) as compare to control. #,### Significant difference (p<0.01, p<0.0001) as compare to diabetic rats.

In alloxan-diabetic rabbits, HbA_1_c and fructosamine levels were considerably higher than in control rabbits (Fig 2A and 2B); eight weeks treatment with aminoguanidine produced significant (p<0.0001) reduction in HbA_1_c and fructosamine contents. The findings suggested that AG ameliorated Amadori product formation.

### Biomarkers of antioxidant defenses, peroxidation and oxidative stress

**Fig 3:**
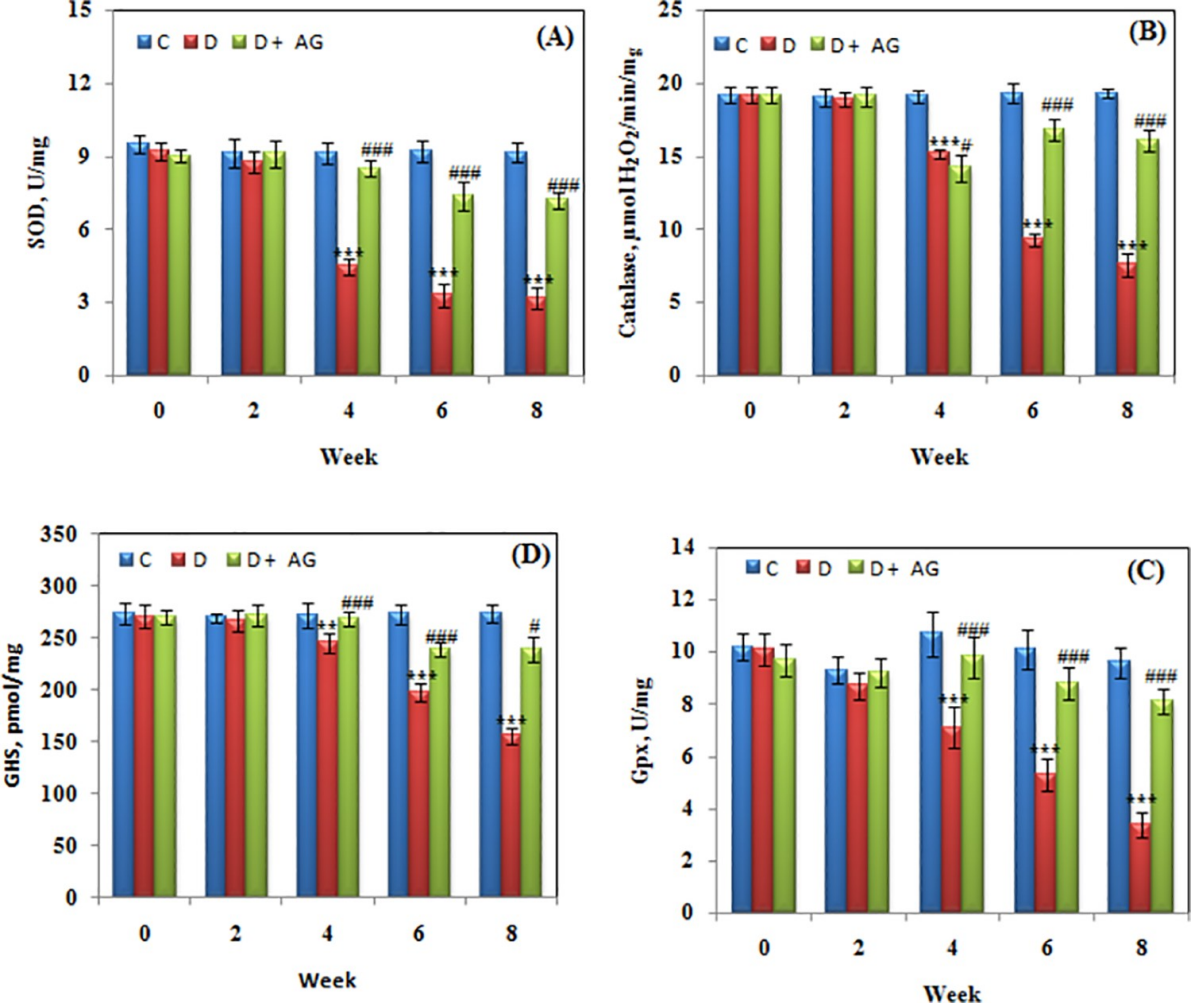
Antioxidant biomarkers of alloxan-diabetic rabbits treated for 8 weeks with aminoguanidine. Activities of SOD (**A**), Catalase (**B**), Gpx (**C**), and level of glutathione (**D**). Values are mean ± SD, (n = 6 rabbits/group). *** Significant difference (p<0.0001) as compare to control. #,### Significant difference (p<0.01, p<0.0001) as compare to diabetic rats.

**Fig 4:**
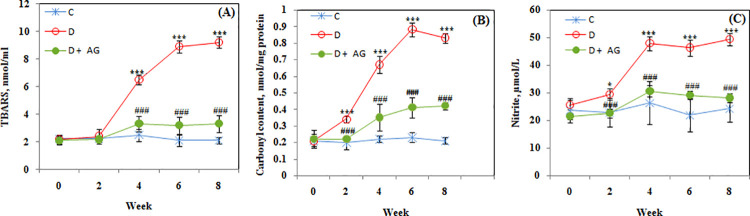
Glycoxidated stress biomarkers of alloxan-diabetic rabbits treated for 8 weeks with aminoguanidine. Levels of TBARS (**A**), Carbonyl (**B**) and nitrite (**C**). Values are mean ± SD, (n = 6 rabbits/group). *,*** Significant difference (p<0.01, p<0.0001) as compare to control. ### Significant difference (p<0.0001) as compare to diabetic rats.

Superoxide dismutase activity was significantly (p <0.0001) reduced in alloxan-diabetic rabbits. Fig 3A illustrates the bi-weekly monitoring of SOD activity in different groups. During eight weeks period the average SOD activity in control group was 9.1±0.41U/mg. In diabetic group the SOD was found to be significantly (p <0.0001) decreased after second week. The AG treatment restored the SOD activity to the extent of 80% during eight week period. The effect of AG on catalase restoration was almost similar to SOD (Fig 3B). The glutathione peroxidase, glutathione, TBARS, carbonyl and nitrite were measured in all three groups. While the first two biochemicals were found to be lower in alloxan-diabetic rabbits than the control group; the TBARS, carbonyl and nitrite were higher in the diabetic group. Furthermore, in alloxan-diabetic rabbits, the TBARS began to rise after second week, while carbonyl and nitrite were already on the higher side at second week. Analysis of glutathione peroxidase (Fig 3C), reduced glutathione (Fig 3D); TBARS, carbonyl and nitrite levels (Fig 4A–4C) in different groups showed significant improvement in these stress-related parameters of animals. The data reiterates that AG is quiet effective in restoring antioxidant defenses by decreasing stress.

**Fig 5:**
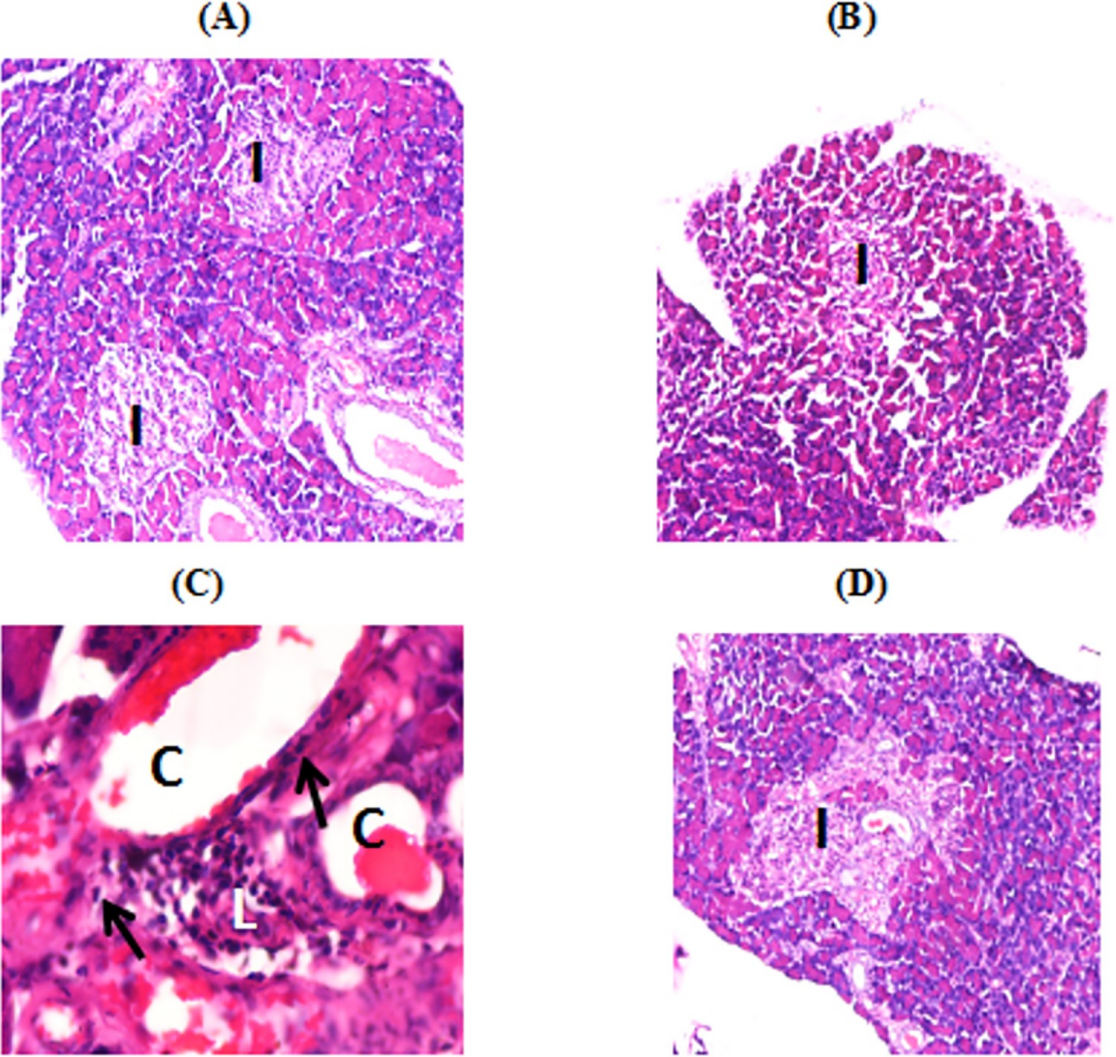
Micrographs of pancreatic tissues of different groups of rabbits. (**A**) Control rabbit pancreas showing normal appearance with islet with a central core of beta cells (I) enveloped by a peripheral, largely continuous mantle of non beta cells. Haematoxylin & Eosin x 10X. (**B&C**) Diabetic group pancreatic tissues showing congestion (C), fibrosis (↑) and perivascular infiltration of lymphocytes (L) and decreased pale islet cells (I). Haematoxylin & Eosin x 10X. (**D**) Aminoguanidine treated diabetic rabbits showing near normal appearance of islet with a central core of beta cells (I). Haematoxylin & Eosin x 10X.

Sections of the pancreas of control animals examined under light microscope showed normal pancreatic architecture (Fig 5A). In contrast, semi-thin sections of pancreas of alloxan-diabetic rabbits revealed islets that were relatively small, atrophied and showed reduction in the number of polygonal islet cells (Fig 5B and 5C). Extensive fibrosis and peri-vascular stromal infiltration of lymphocytes & congested blood vessels was observed. The diabetic animals treated with AG revealed lightly stained elongated islets of similar size as observed in the control animals (Fig 5D).

## Discussion

Chronic hyperglycemia in diabetics may cause extensive non-enzymatic glycation of proteins leading to early- and advanced-glycation end products. Poor metabolic control and brief episodes of hyperglycemia have been linked to diabetic complications, resulting in a phenomenon known as "metabolic memory," which causes significant alterations in numerous tissues. Metabolic memory refers to the persistence of diabetic issues after metabolic control has been achieved; it is the result of a number of mechanisms, including the production of AGEs, oxidative stress, inflammation, and epigenetic alterations [36]. Diabetics as well as experimental models of DM may exhibit oxidative stress due to accelerated generation of ROS that depletes the activity of antioxidant defense mechanisms [37]. Excessive generation of free radicals accompanied by a decrease in the antioxidant capacity may lead to irreversible changes in tissues. Therefore, preventing the non-enzymatic early glycation of relevant proteins or blocking their subsequent effects may be helpful in preventing the complications of DM.

The alloxan-induced type1 DM model is well-established in rabbits; the chemical causes severe necrosis of pancreatic β-cells [38]. With the generation of superoxide radicals, alloxan and its reduction product, dialuric acid, start a redox cycle. The radicals are then dismutated into hydrogen peroxide. The Fenton reaction then produces highly reactive hydroxyl radicals. Alloxan’s cytotoxic effect on β-cells is thus mediated via the production of superoxide and hydroxyl radicals [39]. In this study, high blood glucose level reiterates the efficacy of alloxan in producing experimental model of type1 DM. However, AG treatment was found to be beneficial in experimental hyperglycemia. This may be attributed to the superoxide and hydroxyl radicals scavenging by AG.

It has been reported that majority of the glycated proteins in plasma exist as Amadori-proteins [40]. The concentration of Amadori-products exceeds that of AGEs not only in serum but also in protein preparations that contain AGEs [6, 41]. Therefore, it is plausible to think that many of the effects that have been ascribed to AGEs may be actually due to Amadori-products. In diabetic patients, HbA_1c_ is both a diagnostic as well as prognostic marker [42]. Moreover, it is also an indicator of glycation status of other plasma proteins [43]. The significant decrease in Amadori-products (HbA_1_c and fructosamine) in AG treated diabetic animals suggested that AG is capable of inhibiting the early glyction products formation. A similar observation has been also reported by Lima et al. [23]. It may be recalled that AG is an established inhibitor of AGEs. Earlier studies have suggested that AG inhibits the glycation by limiting the attachment of glucose with amino groups of the proteins [44–46]. Another study reported that AG might be interacting with the carbonyl groups of early glycation products forming stable adducts that do not react further [47].

It has been widely held that oxidative stress may constitute the key and common events in the pathogenesis of DM complications. Both enzymatic and non-enzymatic antioxidants play an important role in free radicals neutralization. During hyperglycemia increased glycation accelerates free radicals generation concomitantly. Together (or individually) they may affect enzymes; especially those with antioxidant properties. For example, SOD is inactivated by glycation of its specific lysine residues that can suppress its antioxidant behavior [48].

The hyperglycemia-increased glycation-increased free radical production triad may be responsible for the decrease in SOD, catalase, GPx, and rGSH levels in alloxan-diabetic rabbits. HbA1c, fructosamine, and MDA levels were reduced by AG therapy, while SOD, catalase, GPx, and rGSH levels were dramatically restored. While discussing similar results of AG alone or in combination with curcumin on streptozotocin-diabetic rats, Lima et al. [23] suggested triggering of the cytoprotective machinery involved in the elimination of ROS and Amadori-product products/intermediates; a similar phenomenon may have occurred in our case as well. El-Missiry and El-Gindy [49] found a link between increased lipid peroxidation and lower levels of antioxidant enzymes in DM. Researchers have discovered that rGSH depletion occurs before lipid peroxidation is induced [50]. AG has been proven to reduce MDA [24] which is consistent with our findings.

Increased production of nitric oxide in early stage of DM has been reported [51, 52]. In this study, diabetic animals showed significant increase in NO level; may be due to tissue injury. AG administration significantly reduced the NO level. The beneficial action of AG as free-radical scavenger might directly eliminate peroxynitrite [17] a product of NO and superoxide anion radical. NO as a free radical is relatively unstable in oxygenated solutions where it rapidly and spontaneously reacts with molecular oxygen to yield a variety of nitrogen oxides. The only stable products formed by spontaneous decomposition of NO in oxygenated mediums are nitrites and nitrates, which are considered to be indicators of NO production [53].

Pancreatic tissues of the alloxan-dibetic rabbits examined under light microscope showed degranulation, degeneration, islet cell necrosis accompanied by peri-vascular infiltration of lymphocytes. However, the islet cell integrity was maintained in AG treated diabetic rabbits. From the results it may be derived that AG has prevented the oxidative modification of cells’ proteins and lipids via antioxidant action and/or by trapping reactive breakdown products of lipid peroxidation (such as aldehydes).

Free radical driven Amadori formation during diabetic hyperglycemia is a critical stage in the progression of complications of the diabetes mellitus. Our data suggest that AG is quite effective in slowing down the formation of Amadori adducts mainly by decreasing of stress and restoration of antioxidant defenses.
